# Contralateral Ovarian Metastasis of Clear-Cell Renal Carcinoma: A Rare Case Report and Review of the Literature

**DOI:** 10.1155/2017/9849205

**Published:** 2017-05-08

**Authors:** Fatih Uruc, Serkan Akan, Aytaç Sahin, Caglar Yildirim, Ahmet Urkmez

**Affiliations:** ^1^Department of Urology, Fatih Sultan Mehmet Research & Training Hospital, Istanbul, Turkey; ^2^Department of Urology, Haydarpasha Numune Research & Training Hospital, Istanbul, Turkey

## Abstract

Renal cell carcinoma (RCC) constitutes 2-3% of all types of cancers. RCCs metastasize into lungs (50–60%), lymph nodes (36%), bones (30–40%), liver (30–40%), and brain (5%) in respective percentages. RCC rarely metastasizes into ovary. Only 25 cases of ovarian tumor, which metastasized into kidneys, have been presented. In the literature, a kidney-ovary axis has been defined, and its interrelationship begins with embryological life. With this case report, we aimed both to present a very rarely seen metastasis of RCC into contralateral ovary and also to review the literature.

## 1. Introduction

Generally metastatic lesions of ovaries originate from gastric and colon and breast carcinomas and lymphomas. RCCs rarely metastasize to ovaries, approximately 6%. Nevertheless some ovarian metastases may be confused with primary ovarian tumor and thus may be overlooked. With this case report, we aimed both to present a very rarely seen metastasis of RCC into contralateral ovary and also to review the literature.

## 2. Case Presentation

**Figure 1:**
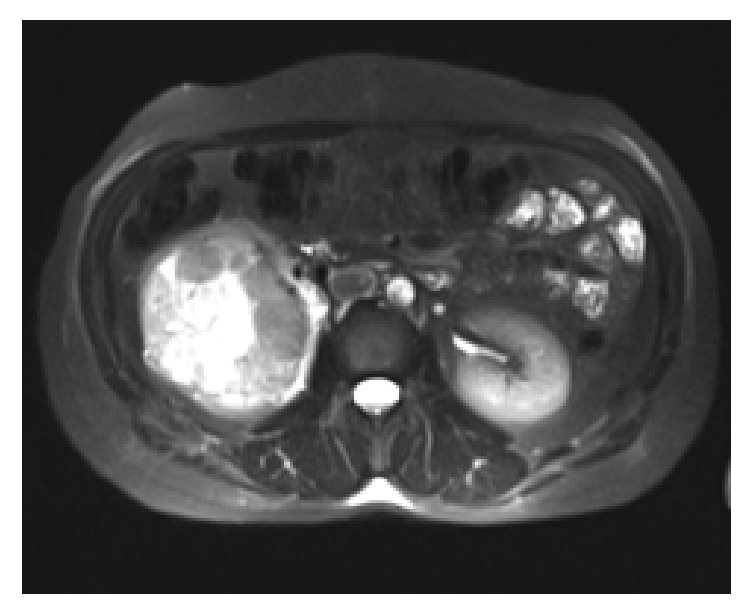
On contrasted CT, 80 mm × 76 mm renal mass with heterogeneous contrast enhancement arising from the lower pole of the right kidney.

A 48-year-old female patient presented to our outpatient clinic with complaints of right flank pain and hematuria lasting for the previous one month. Computed tomographic (CT) examinations performed on the patient with a history of renal calculi revealed 80 mm × 76 mm renal mass arising from the lower middle pole of the right kidney and demonstrating a heterogeneous contrast uptake (Figure 1). Consequent magnetic resonance imaging (MRI) disclosed 81 mm × 77 mm heterogeneous renal mass and invasion to the right renal vein. MRI did not detect intraabdominal and skeletal metastatic foci. Multislice CT also could not reveal any metastatic foci in the lungs.

**Figure 2:**
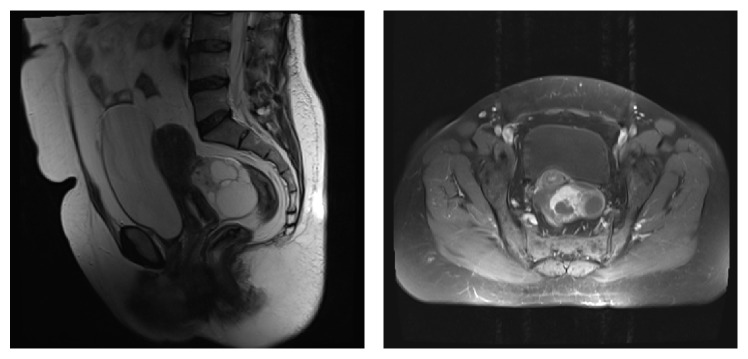
On contrasted MR a lesion measuring nearly 58 mm × 68 mm cyst with an increased signal intensity (30 HU), which fills Douglas recess and extends into the left adnexa. Initially the lesion was thought to be a left ovarian cyst but histopathology report demonstrated a metastatic lesion of renal clear-cell carcinoma with dimensions of 70 mm × 50 mm × 50 mm.

Laparoscopic right radical nephrectomy was performed and a mass measuring nearly 70 mm × 60 mm × 60 mm and more frequently involving middle and lower poles of the kidney was extracted. Histopathology was reported as clear-cell renal carcinoma (Fuhrman Grade I). Capsular invasion and lymphovascular invasion were not seen. Surgical margin negativity was reported. Any pathological finding was not encountered during routine control visits, and imaging modalities (thoracic CT and whole abdominal CT) were performed at postoperative 3, 6, 12, and 18 months. At postoperative 22 months, ultrasonograms obtained because of lower left abdominal quadrant pain disclosed a hemorrhagic cystic mass lesion in the left ovary measuring 40 mm × 30 mm. Tumor biomarkers were within normal limits [*β*-Hcg < 1.2 mIU/mL, CA 125: 34.3 U/mL, CA 15-3: 22.5 U/mL, CA 19-9: 8.3 U/mL, and CEA 2.09 ng/mL]. Cervicovaginal smear cytology results were negative for dysplasia and malignancy. Contrast-enhanced computed tomography revealed a nearly 58 mm × 68 mm cystic mass with high signal intensity (30 HU), which filled the entire Douglas recess, and extended up to the left adnexa which was initially thought to be a left ovarian cyst (Figure 2). The right ovary, myometrium, endometrium, and cervix were of natural appearance.

**Figure 3:**
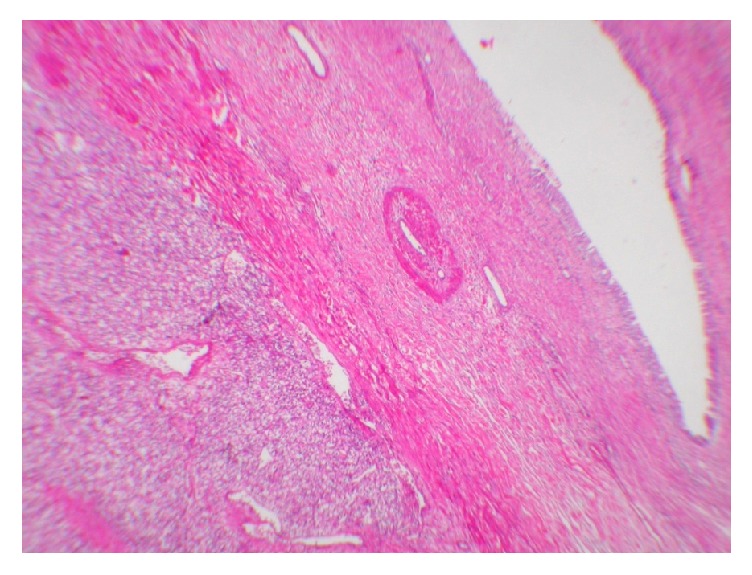
Renal cell carcinoma closer to the pelvic cavity observed under small magnification (H&E ×40).

During laparotomy, which was performed by the department of obstetrics and gynecology, frozen sections were sent from the left ovary, left fallopian tube, and left infundibulopelvic ligament. The cyst had adhesions, irregularities, and malignant appearance and it was larger than previously reported. According to the pathology of frozen section which was reported as suspected malign neoplasm, left salpingo-oophorectomy was performed on her. Histopathology report demonstrated a metastatic lesion of renal clear-cell carcinoma with dimensions of 70 mm × 50 mm × 50 mm, paratubal cyst, and hyperemia (Figure 3). Nearly 2 years ago its nuclear grade was Grade 1, while presently its grade raised to Grade 3. The patient is at her postoperative 24 months after LRN. Imaging modalities performed did not reveal any evidence of intraabdominal, thoracic, and skeletal recurrence or metastatic foci.

## 3. Discussion

RCC recurs especially within the first 3 years in 20–40% of nephrectomized patients because of a clinically localized disease. Kim et al. reported postoperative renal recurrences in nearly 5% and development of metastasis in 15% of the patients operated and followed up with the indication of renal clear-cell carcinoma [1]. Our patient with metastatic RCC (mRCC) is a rarely seen case.

In the literature, a kidney-ovary axis has been defined, and its interrelationship begins with embryological life. In women with renal clear-cell cancers, genital tract RCC metastasizes frequently to vagina [2]. Although metastases to ovaries are relatively rare, metastases of RCC to contralateral ovary are seen more rarely [3]. Metastases of RCC to ovaries can be detected before or years after the diagnosis of primary renal tumor, and they can be confused with primary tumors of ovaries [4].

Metastatic renal tumors more frequently spread through hematogenous route. Drainage of the left ovarian vein to the left renal vein explains this hematogenous spread [5]. This phenomenon rather explains the metastasis of the left RCC to the ipsilateral ovary. However it does not account for the metastasis to the contralateral ovary. Germ cell testicular tumors may spread through thoracic duct to contralateral supraclavicular lymph node and subclavian vein [6]. The most important determinant of this metastasis is the presence of lymphovascular invasion in the primary tumor. Retrograde venous embolization through the renal vein is thought to be involved in the pathogenesis of ovarian metastases of RCC. Close anatomical relationship between renal and gonadal vein and more frequent metastasis to the left ovary which constitutes two-thirds of the cases reported in the literature have made the researchers focus on the abovementioned pathogenesis.

Renal clear-cell carcinoma more frequently metastasizes to ovaries when compared with renal papillary cell carcinoma of which only one case has been reported in the literature so far [4]. In compliance with the literature, histopathology of the primary renal tumor was reported as renal clear-cell carcinoma. Only 6% of RCCs metastasize to ovaries, generally metastatic lesions of ovaries originate from gastric and colon and breast carcinomas and lymphomas. RCC rarely metastasize to ovaries. Some justifications suggested for this rarity may include its 2 times increased incidence in men, development of postmenopausal vascular sclerosis in ovaries in the age bracket at a time where incidence of RCC skyrocketed, and thirdly some ovarian metastases may be confused with primary ovarian tumor and thus may be overlooked.

Glandular metastases of RCC (pancreas, breast, parotid, thyroid, ovary, or contralateral adrenal) are rare in metastatic clear cell. These various metastatic sites that we considered as glandular metastases are infrequent sites of metastasis. Glandular metastases, particularly pancreatic and adrenal metastases, are often associated with good survival in the literature. The characteristics of the glandular metastases clearly differed from those of the nonglandular metastases, with less aggressive characteristics, more frequent Grade I/II, longer delay between kidney tumor and first metastases, and good risk at metastatic presentation. In a recent meta-analysis, 138 patients from 9 European centers (5 French, 3 UK, and 1 Belgian centers) with a diagnosis of RCC with glandular metastasis between the dates of January 2004 and October 2013 were examined and there were no cases with ovarian metastases [7]. However, in our case, survival achieved by surgical treatment of overmetastatic RCC is consistent with the literature on glandular metastases.

Generally metastatic ovarian tumors are unilateral. Lerwill et al. [8] reported the largest metastasis (125 mm in diameter) of RCC to ovary.

Also in our case a 58 mm × 68 mm cystic mass filling the entire Douglas recess and extending up to the left adnexa was initially thought to be a left ovarian cyst and reported as a lesion with a high signal intensity (30 HU). However in consideration of the medical past of the patient instead of watchful surveillance radical surgery was preferred.

## 4. Conclusion

Metastatic ovarian masses are treated using radical surgical procedures just like primary ovarian tumors. Thus it is possible to achieve long-term disease-free survival rates in these patients.
